# Magnetic graphene oxide-nano zero valent iron (GO–nZVI) nanohybrids synthesized using biocompatible cross-linkers for methylene blue removal†

**DOI:** 10.1039/c8ra08386j

**Published:** 2019-01-08

**Authors:** Novin Mehrabi, Arvid Masud, Moyosore Afolabi, Jinwoo Hwang, Gabriel A. Calderon Ortiz, Nirupam Aich

**Affiliations:** Department of Civil, Structural and Environmental Engineering, University at Buffalo, The State University of New York Buffalo NY 14260 USA nirupama@buffalo.edu; School of Civil and Environmental Engineering, Georgia Institute of Technology Atlanta GA 30332 USA; Department of Materials Science and Engineering, Center for Electron Microscopy and Analysis, The Ohio State University Columbus OH 43212 USA

## Abstract

GO and nZVI have been used for removing different contaminants from aqueous solution; however, difficulty in the separation of GO, and the aggregation propensity of nZVI particles prevent them from having efficient practical applications. In this study, a green synthesis method was performed to prepare nanohybrids of GO and nZVI to provide an adsorbent with high adsorption efficiency that can be removed from aqueous solution easily by magnetic separation. GO–nZVI nanohybrids were synthesized by using biocompatible cross linkers named 1-ethyl-3-(3-dimethylaminopropyl)-carbodiimide (EDC) and *N*-hydroxysuccinimide (NHS). The effect of the nZVI ratio in the synthesized nanohybrids was studied at three different ratios of GO : nZVI, 1 : 1, 1 : 5 and 1 : 10. SEM/EDS, HRTEM, STEM/EDS, XRD, Raman, FTIR, and TGA analyses were conducted to provide physical and chemical properties of the adsorbents. The performance of nZVI and GO–nZVI nanohybrids as an adsorbent have been studied for methylene blue (MB) removal from an aqueous solution with an initial concentration of 12 mg L^−1^ at adsorbent dosages of 0.1, 0.3, 0.5, and 1 mg mL^−1^. Results indicated that GO–nZVI (1 : 5) provided the highest MB removal (99.1%) by using 10 mL of the 1 mg mL^−1^ adsorbent. After regeneration of the GO–nZVI (1 : 5) nanohybrids with ethanol, 84.3%, 67.2%, and 63.0% of MB removal were achieved in the first to third regeneration cycle. Results also showed that the GO–nZVI nanohybrids were not affected by aggregation compared to nZVI.

## Abstract 2

A green synthesis method was used to prepare GO–nZVI nanohybrids to provide an adsorbent with high adsorption efficiency that can be removed from aqueous solutions easily by magnetic separation.

## Graphical abstract

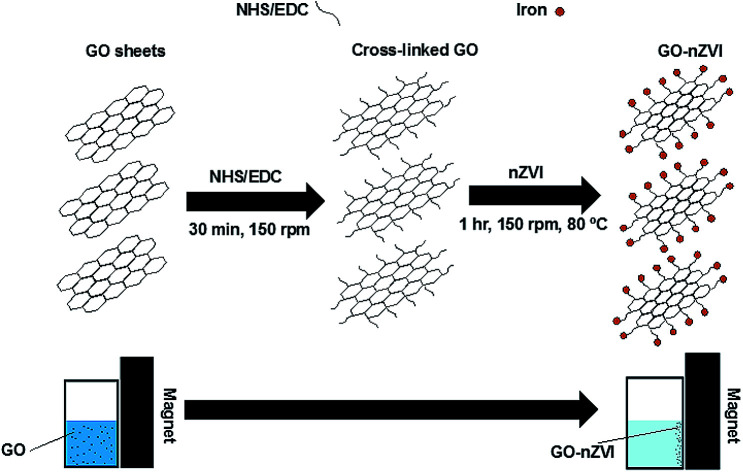

## Introduction

Nano-scale zero valent iron (nZVI; Fe^0^) particles have attracted a lot of attention, especially in environmental applications mainly due to their large surface area and high reactivity.^1^ nZVI is the most used nanomaterial in the world for environmental applications, especially as nZVI has been used in the United States and Europe for contaminated soil and groundwater remediation and recently in the industrial wastewater treatment processes.^2,3^ nZVI can be oxidized to Fe^2+^ or Fe^3+^ and donate electrons that can effectively adsorb and/or reduce organic and inorganic contaminants as well as heavy metals. Furthermore, nZVI's magnetic properties allow for its easy separation and recovery from the waste stream for regeneration and reuse. However, nZVI suffers significantly from aggregation due to its high van der Waals and magnetic attraction forces; this leads to a decrease in pollutant removal efficiency by the decline in reactive surface area.^4^

Aggregation of nZVI particles can be prevented either by modifying their surface using surfactant and polymers, or by loading these nanoparticles on a solid support.^5^ Different supports ranging from macro to micro and recently nanoscale solid materials have been pursued which include zeolites, clay, biopolymers, as well as carbon-based materials such as activated carbons, carbon nanotubes, and graphene oxide. Compared to the activated carbon and carbon nanotubes, graphene and its derivatives due to their planar two-dimensional nanostructure provide extended surface area allowing for the growth of individual nZVI particles with less aggregation, high electrical conductivity, and mechanical durability.^5^ Furthermore, GO has shown better adsorption property than carbon nanotubes and activated carbons against organic contaminants^6^ and heavy metals.^7^ Such excellent adsorption properties of GO are attributed to the hydrophobic interactions with the large extended carbon plane in case of organic contaminants, and to the electrostatic interactions with the carboxyl, hydroxyl, or carbonyl groups present on the GO surface in case of heavy metals. Thus, while nZVI particles are deposited on graphene-based materials, usually reduced graphene oxide (rGO), the nanohybrids exhibit superior performances in removing contaminants due to a combined enhancement of the adsorptive and reductive capacities.^8^ Such enhancement of contaminant removal ability of rGO–nZVI nanohybrids have accelerated the research on their various synthesis procedures.

While synthesis of multicomponent carbon–metal nanohybrids (*e.g.*, rGO–nZVI or GO–nZVI) has become attractive for exploiting the synergistic activities of the component materials to enhance the desired functionality or to achieve the multifunctional properties; unique property alterations and emergent new properties in the nanohybrids (compared to the parent materials) can lead to uncertain fate and toxicity upon exposure to the environment.^9^ Thus, there is a need of employing safer-by-design approach for synthesizing multifunctional next generation of nanohybrids. Typical synthesis methods for graphene supported nZVI include chemical reduction, chemical vapor deposition (CVD), sonochemical treatment, hydrothermal or solvothermal processes, plasma-based technique, and spray pyrolysis – all of which essentially produce rGO–nZVI from GO and iron salt precursors.^5^ Many of these processes use various harmful reducing agents including sodium borohydride, hydrazine, and benzene. Most of these conventional synthesis methods co-reduce GO while depositing nZVI particles from iron salt precursor on rGO support. However, GO exhibits higher contaminant removal capacity than rGO for certain specific contaminants *e.g.*, heavy metals,^10^ cationic dyes.^11^ Furthermore, while nZVI is deemed to be non-toxic due to their high aggregation propensity, it is unknown how their toxic behavior would change upon the formation of rGO–nZVI nanohybrids. This becomes particularly important since rGO has shown to be more toxic to aquatic organisms^12,13^ and mammalian cells^14^ when compared to GO. Our recent study has shown that rGO–nZVI, synthesized by the most commonly used chemical co-reduction technique, shows higher toxicity than nZVI (data not shown).^15^ Thus, safer strategies to conjugate nZVI onto graphene supports need to be pursued.

This study presents a new method of conjugating pre-synthesized nZVI onto GO nanosheets using a biocompatible crosslinker – a technique that has the potential to create safer GO–nZVI nanohybrids. Crosslinkers can conjugate materials to each other based on the presence of particular moieties that are able to react with the specific kinds of functional groups.^16^ Here we used well-known carbodiimide crosslinker chemistry using 1-ethyl-3-(3-dimethylaminopropyl)-carbodiimide hydrochloride (EDC) and *N*-hydroxysuccinimide (NHS) crosslinking agents. EDC-NHS coupling chemistry has been prevalent majorly in drug delivery and nanomedicine literature^17–20^ due to their excellent biocompatibility and also the ability for nanoparticle surface modification and conjugation. EDC can easily bond with the carboxylic acid groups of GO and then can lead to esterification upon addition of NHS. nZVI has a high affinity for amine functional groups^21,22^ and thus will probably attach to the NHS, providing a GO–nZVI hybrid linked *via* EDC-NHS chain. This well-established EDC-NHS coupling chemistry has been previously used in conjugating iron oxide nanoparticles with graphene for enabling contaminant removal; however, those involve surface modification of iron oxide nanoparticles using amine functional groups.^23,24^ Furthermore, no reports of using EDC-NHS crosslinkers have been reported for conjugating nZVI directly on the GO surfaces to synthesize GO–nZVI nanohybrids.

In this study, GO and nZVI without any other surface modification, for the first time, were conjugated using biocompatible EDC-NHS crosslinkers to synthesize GO–nZVI nanohybrids. Three different sets of GO–nZVI nanohybrids were synthesized using different precursor ratios *i.e.*, GO : nZVI ratio of 1 : 1, 1 : 5, and 1 : 10. Physical and chemical properties of the synthesized nanohybrids were characterized by high-resolution transmission electron microscopy (HRTEM), scanning electron microscope (SEM), energy dispersive X-ray spectroscopy (EDS), scanning transmission electron microscopy coupled with energy dispersive X-ray spectroscopy (STEM/EDS), X-ray diffraction (XRD), thermogravimetric analysis (TGA), and Raman and Fourier transform infrared (FTIR) spectroscopy. The ability of the prepared GO–nZVI nanohybrids to remove contaminants from aqueous media was evaluated by performing removal of methylene blue (MB) dye and was compared to the MB removal ability of nZVI only.

## Material and methods

### Preparation of GO–nZVI nanohybrids

Single layer graphene oxide (GO, > 99.3 wt%, thickness 0.43–1.23 nm) and nZVI (99.5%, 25 nm) were purchased from US Research Nanomaterials, Inc (Houston, TX). First, 50 mg of GO was dispersed in 100 mL of deionized (DI) water using a microtip-based ultrasonic dismembrator (Qsonica, Newtown, CT) for 3 h on pulse-mode (5 min on, 1 min off) at an amplitude of 50 and input power of 15–18 W. The sonication was performed under temperature control using ice bags (15 °C). Then, the suspension was mechanically stirred at 150 rpm using an open drum mixer (Dayton Inc.) while 25 mg of EDC (BioXtra) and 20 mg of NHS (98% purity), both purchased from Sigma Aldrich (St. Louis, MO), were slowly added to the suspension. The obtained suspension was mixed for 30 min at ambient temperature. Then, 50, 250, or 500 mg of nZVI was added to the suspension for synthesizing GO–nZVI nanohybrids with different ratios GO : nZVI of 1 : 1, 1 : 5, and 1 : 10, respectively. The suspension was kept mixing for 1 h at 80 °C using an open drum mechanical mixer to avoid the possibility of magnetic attraction during the nanohybrid synthesis. Then, the suspension was filtered through a 0.22 μm polyvinylidene fluoride (PVDF) filter (Millipore, Bedford, MA), and the solid residue was washed five times with ethanol and DI water. Finally, solid residue on the filter paper was dried at 65 °C in an oven before further use, and the solid final products were named GO–nZVI (1 : 1), GO–nZVI (1 : 5), and GO–nZVI (1 : 10) based on corresponding precursor ratios.

### Physicochemical property characterization of GO–nZVI nanohybrids

High-resolution TEM (HRTEM) images of GO, nZVI, GO-NHS/EDC, and GO–nZVI nanohybrids were obtained using a JEOL JEM 2010 transmission electron microscope (JEOL, Peabody, MA) operating at 200 kV to determine the physical morphology of the synthesized nanohybrids and the parent nanomaterials. In order to prepare the TEM samples, 1 mg of each sample was sonicated in 20 mL of ethanol (100%) for 15 min, then two drops of the suspensions were placed on carbon-coated copper grids (200 mesh, SPI supplies, West Chester, PA), and left for 20 min to dry before placing on the TEM sample holder. Microscale morphologies and elemental composition of the samples were characterized using a Hitachi SU70 Field Emission Scanning Electron Microscope (FESEM, Hitachi, Japan) at 20 kV coupled with an Oxford energy dispersive spectrometer (EDS, Oxford Instruments, Concord, MA). The nanomaterial samples for SEM/EDS characterization were placed on a stub covered with double-sided adhesive carbon tape. Then, a thin layer of gold (∼20 nm) was deposited on the samples using DV-502 thin film deposition system (Denton Vacuum, Moorestown, NJ). More accurate quantification of the chemical composition along with nanoscale morphology characterization was performed using highly advanced Tecnai G2 30 TWIN scanning transmission electron microscope coupled with an energy dispersive X-ray spectroscope (STEM/EDS) operated at 300 kV. This STEM/EDS system was used to acquire the high angle angular dark field (HAADF) images and EDS maps. EDS maps were obtained for 20 minutes per frame using an EDAX TEAM silicon drift detector in the STEM. Brightness and contrast were adjusted with no major image alterations. The same TEM grids prepared for HRTEM imaging were used for STEM/EDS analyses.

10 mg of the nanohybrids was dispersed in 10 mL of DI water *i.e.*, in aqueous media for 5 min using a bath sonicator (Branson, 2800, Danbury, CT) to mimic a condition similar to adsorbent usage for MB removal. A similar dispersion of nanohybrids was sonicated for 1 h (8 s on, 2 s off) at high energy using a microtip based ultrasonicator (Qsonica, Newtown, CT) at an amplitude of 50. In both cases, after the sonication, the nanohybrids were separated using the magnet, and the iron content of the remaining aqueous media was measured using an Inductively Coupled Plasma Optical Emission Spectrometer (ICP-OES, iCAP 6000, Thermo Scientific, Waltham, MA).

The magnetic properties of the nZVI and GO–nZVI nanohybrids were measured at room temperature by a vibrating sample magnetometer (VSM, Evercool II, Quantum Design, San Diego, CA).

ULTIMA IV X-ray diffractometer (XRD, Rigaku, Wilmington, MA) equipped with Cu laser source was used to characterize the crystal structures and phases of the samples. The powdered samples were placed on a glass slide and then put inside the XRD sample holder before scanning at a rate of 2° min^−1^ within a range from 5° to 90°. Vibrational frequencies and structural changes of the samples were obtained using a Raman microscope (InVia Qontor, Renishaw, West Dundee, Illinois) with an incident laser wavelength of 514 nm. Functional groups of the samples were characterized using a Fourier-transform infrared (FTIR) spectroscopy instrument (1760 FTIR, Perkin-Elmer, Waltham, Massachusetts) with 4 cm^−1^ resolution within the range of 400–3500 cm^−1^. The samples' thermal durability was studied by performing a thermogravimetric analysis (Q600 STD, TA Instrument, Norcross, GA) under nitrogen purging and with a heating rate of 10 °C min^−1^ from room temperature up to 900 °C.

### Methylene blue (MB) removal

A stock solution of MB (Fisher Scientific, Waltham, MA) in DI water with the initial concentration of 12 mg L^−1^ was prepared. Each 10 mL MB solution was poured in a 20 mL vial, where nano-adsorbents *i.e.*, the three different sets of GO–nZVI nanohybrids or bare nZVI at different amounts (1, 3, 5, or 10 mg) were added to the MB solution. Then, the mixture was bath sonicated (Branson, 2800, Danbury, CT) for 5 min and after that, the nano-adsorbents were separated using a simple ceramic block magnet with dimensions of 0.38 × 0.88 × 1.88 in^3^ (Master magnetics, Castle Rock, CO). The concentration of MB solutions after each adsorption test was determined by measuring their light absorbance values at 665 nm wavelength using a Cary 60 UV-vis spectrophotometer (Agilent, Santa Clara, CA), and the obtained results were compared with the absorbance of the prepared reference MB solutions of known concentrations. MB concentration in each case was computed based on a calibration curve prepared earlier following the Beer–Lambert law which states that the concentration of the solution and the light absorbance values are linearly proportional. Each of the adsorption experiments was performed in triplicates, and the average values of removal efficiency along with standard deviation were calculated.

### Adsorbent regeneration and reuse

After the first set of adsorption experiments, the adsorbent with the best performance *i.e.*, the highest MB removal was selected for performing regeneration and reuse. After the MB adsorption experiment was performed and the adsorbents were magnetically separated, the remaining MB solution in the vial was decanted while keeping the adsorbent separate using the magnet. Then, 10 mL pure ethanol was added to the vial, magnet was taken away, and the mixture of adsorbent and ethanol was bath sonicated for 5 min. Then the adsorbents were separated using the magnet, and ethanol was decanted to retrieve the regenerated adsorbents for further reuse in MB removal. Three regeneration and reuse cycles were performed to determine the recyclability of the adsorbent. The regeneration steps are presented using a schematic briefly (Fig. S1†).

## Results and discussion

### Physical morphology and chemical composition of GO–nZVI nanohybrids

**Fig. 1:**
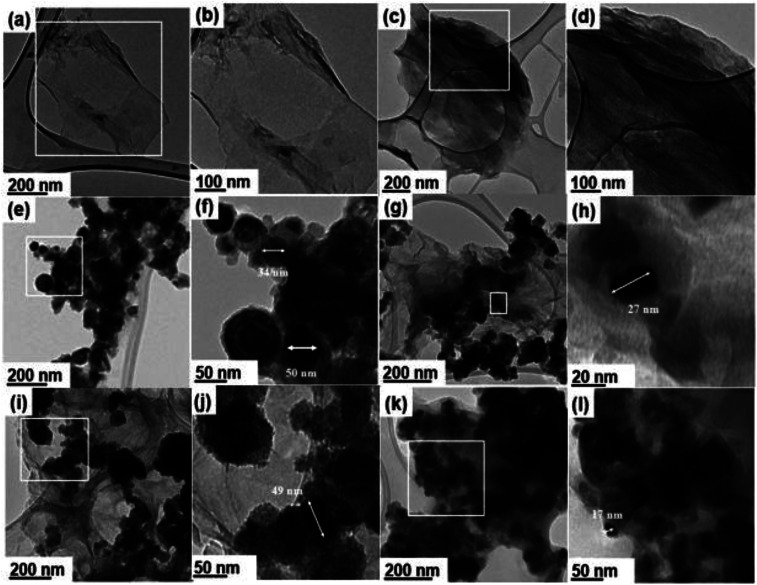
HRTEM micrographs of GO (a and b), GO-NHS/EDC (c and d), nZVI (e and f), GO–nZVI (1 : 1) (g and h), GO–nZVI (1 : 5) (i and j), and GO–nZVI (1 : 10) (k and l).

Fig. 1 presents HRTEM micrographs of different samples including the synthesized GO : nZVI nanohybrids and their component materials. Each sample set has two HRTEM images – first one at low and the second one at high magnification. Fig. 1a and b show single to few layered planar two-dimensional GO nanosheets with lateral size in the range of 0.7 to several microns, which become quasi-transparent under HRTEM. Fig. 1c and d show the HRTEM images of GO after adding NHS/EDC and before addition of nZVI. These images indicate that multiple layers of GO nanosheets aggregated with each other, possibly due to the cross-linking by NHS/EDC. Fig. 1e and f show the HRTEM images of bare nZVI, while Fig. 1g–l present the micrographs of GO–nZVI nanohybrids in a sequence of low-to-high nZVI content. Furthermore, individual nZVI particles were analyzed for their particle size distribution in each of the samples including bare nZVI and GO–nZVI nanohybrids, and the corresponding results have been plotted as histograms in Fig. S2 in the ESI.† Bare nZVI particles were spherical and formed a chain-like agglomerated structure in which each particle has a dark core surrounded by a thin oxide shell as seen in Fig. 1e and f-consistent with previous studies.^25,26^ Although some of the bare nZVI aggregates had sizes close to ∼100 nm, about 90% of the particles had sizes in the range of 19–65.8 nm. From the image analyses, the average Martin diameter (with standard deviation) of bare nZVI particles was found to be 38.6 ± 17.1 nm – a value consistent with previous studies.^27,28^Fig. 1g–l show the successfully conjugated nZVI on the GO surfaces for GO–nZVI nanohybrids with GO : nZVI ratios of 1 : 1, 1 : 5, and 1 : 10, respectively. The mechanism behind the conjugation is probably more of a chemical in nature rather than physical one, since the conjugates remained intact even after high energy sonication in ethanol for 15 minutes before the TEM grid preparation. This has been further explored by examining the elemental map images obtained by EDS mapping, and the functional groups and bonding characteristics of the samples obtained by FTIR measurements. The increase in nZVI content in the GO–nZVI nanohybrids leads to more nZVI accumulation and agglomeration on GO surfaces. The average nZVI particle sizes were found to be 28.6 ± 10.6, 32.6 ± 8.0, and 36.6 ± 13.3 nm for GO–nZVI (1 : 1), (1 : 5), and (1 : 10), respectively. Thus, in case of GO–nZVI nanohybrids with lower nZVI content (*i.e.*, when GO : nZVI is 1 : 1 or 1 : 5), the nZVI particle sizes are significantly smaller than bare nZVI particles and were less aggregated due to their attachment on dispersed sites on GO surface. However, with more nZVI content in case of GO–nZVI (1 : 10), the GO surface is completely covered and/or overwhelmed with the nZVI, which leads to high extent of nZVI aggregation and provides particle sizes close to those of bare nZVIs similar to the previous studies.^29,30^

EDS analysis coupled with SEM imaging was first used to examine the gross elemental compositions of the samples at the microscale. EDS spectra showing the elemental distribution of the samples along with corresponding SEM images featuring their bulk morphology are provided in Fig. S3.† For the parent materials, EDS spectra showed the presence of carbon and oxygen atoms for GO (Fig. S3a†), and the presence of iron and oxygen atoms for nZVI (Fig. S3b†). EDS spectra of GO–nZVI nanohybrids presented in Fig. S3c–e† showed the presence of iron, carbon, oxygen, and nitrogen in each of the samples, providing further approval of successful hybridization at the microscale.

**Fig. 2:**
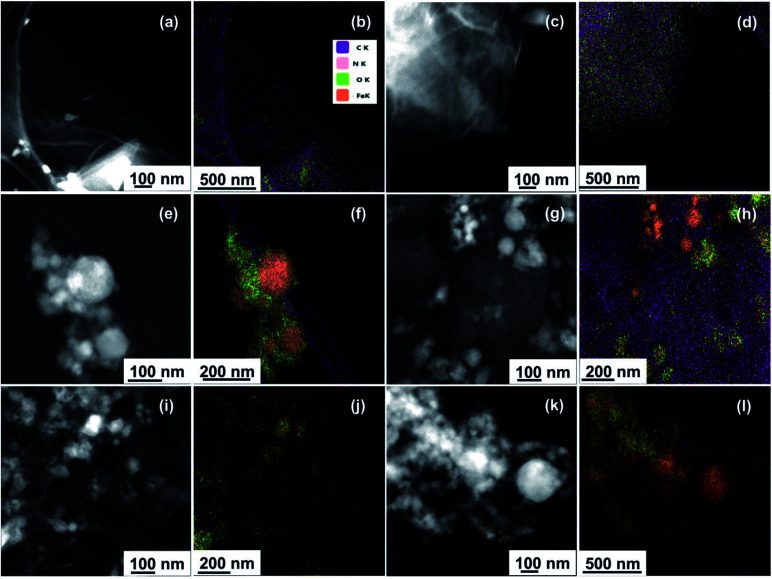
STEM/EDS mapping of GO (a and b), GO-NHS/EDC (c and d), nZVI (e and f), GO–nZVI (1 : 1) (g and h), GO–nZVI (1 : 5) (i and j), and GO–nZVI (1 : 10) (k and l).

Moreover, the peak intensity for iron in the EDS spectra increased with increasing nZVI loading in the nanohybrids. To further confirm and quantify the elemental compositions of the samples at the nanoscale, STEM microscopy was performed to obtain high angle annular dark field (HAADF) images along with EDS mapping. HAADF images for GO, GO-NHS/EDC, nZVI, and GO–nZVI (1 : 1, 1 : 5, and 1 : 10) are presented respectively in Fig. 2a, c, e, g, i and k, and corresponding overlayed elemental maps are presented in Fig. 2b, d, f, h, j and l. Fig. S4a–f† present with EDS maps of each individual element (C, N, O, and Fe) in each sample. EDS map of GO-NHS/EDC (Fig. 2d) shows that after crosslinking of GO with NHS/EDC, nitrogen is added to the sample indicating the successful crosslinking. EDS maps of GO–nZVI nanohybrids showed the presence of carbon, oxygen, nitrogen, and iron indicating the successful functionalization and conjugation. The chemical composition (wt%) of GO, GO-NHS/EDC, nZVI, and GO–nZVI nanohybrids obtained from STEM/EDS mapping are presented in Table 1. As can be seen, by increasing the nZVI content in the nanohybrids, iron content increases which is consistent with the HRTEM images. GO–nZVI (1 : 1, 1 : 5, and 1 : 10) nanohybrids had iron contents of 26.5%, 38.5%, and 72.6%, respectively. Furthermore, detected carbon content (8.9%) in GO–nZVI (1 : 10) was significantly lower than carbon contents (54% and 33.9%, respectively) of GO–nZVI (1 : 1) and GO–nZVI (1 : 5) showing that GO substrate is mostly covered with iron at high iron content. Similar results of elemental distribution for graphene-iron nanohybrids have been reported previously.^28,31,32^

**Table 1:** Chemical composition of GO, GO-NHS/EDC, nZVI, and GO–nZVI nanohybrids obtained from STEM/EDS mapping

Samples	Composition (wt%)					
	GO	GO-NHS/EDC	nZVI	GO/nZVI (1 : 1)	GO/nZVI (1 : 5)	GO/nZVI (1 : 10)
C	70.85	57.73	4.81^a^	54.05	33.87	8.92
O	29.15	40.67	8.79	18.18	27.03	18.43
N	—	1.59	—	1.26	0.58	0.02
Fe	—	—	86.40	26.51	38.52	72.63

a Due to the carbon film of the TEM grid.

The stability of the nanohybrids was assessed by measuring the released iron ion from the nanohybrids after 5 min of bath sonication (representing adsorbent usage condition) and 1 h of high-energy microtip sonication in aqueous media (representing an extreme condition). Results indicated that at adsorption representing condition of 5 min bath sonication, the concentration of iron released to the aqueous media reached 0.004 and 0.012 ppm for GO–nZVI (1 : 5) and GO–nZVI (1 : 10), respectively. At extreme condition of 1 h microtip sonication, the concentration of iron released to the aqueous media reached 0.151 and 0.233 ppm for GO–nZVI (1 : 5) and GO–nZVI (1 : 10), respectively. Based on these results, the amounts of the released iron from the nanohybrids are negligible at adsorption relevant conditions. Although after sonicating of the nanohybrids at extreme conditions during high energy microtip sonication, the released amounts of iron increased significantly compared to the bath sonication, the amounts are still lower than 0.3 ppm which is the maximum contaminant limit of iron in the drinking water.^33^ Therefore, it can be concluded that the synthesized nanohybrids have a good stability, and may not cause concerns about environmental health and safety issues while being used as an adsorbent.

Fig. S5† shows the magnetization-hysteresis loops of the GO–nZVI nanohybrids and nZVI at room temperature. The saturation magnetization (SM) values obtained from the analyses of the magnetization-hysteresis loops for the nZVI, GO–nZVI (1 : 5), and GO–nZVI (1 : 10) nanoparticles were found to be 102.3, 41.9, 47.6 and emu g^−1^, respectively, which refers to high magnetic attraction for all of the samples. These values are in agreement with previous literature reports^34–36^ and guaranteed successful magnetic separation of the nanoparticles after adsorption. The magnetic property of the nanohybrids is lower than the pure nZVI because of the presence of GO. As expected, GO–nZVI (1 : 10) shows higher SM compared to GO–nZVI (1 : 5).

### Crystalline structure of GO–nZVI nanohybrids

**Fig. 3:**
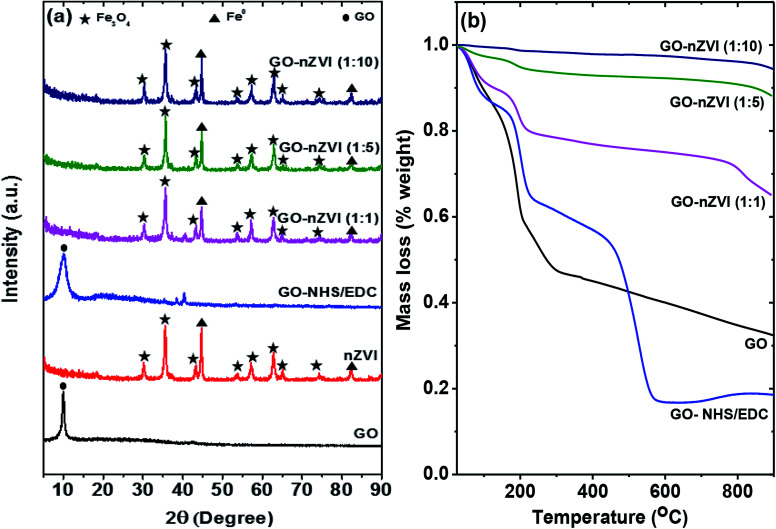
(a) X-ray diffraction (XRD) spectra of GO, nZVI, GO-NHS/EDC, GO–nZVI (1 : 1), GO–nZVI (1 : 5), and GO–nZVI (1 : 10), and (b) TGA curves of GO, GO-NHS/EDC, GO–nZVI (1 : 1), GO–nZVI (1 : 5), and GO–nZVI (1 : 10).

Fig. 3a presents the XRD patterns of the GO, GO-NHS/EDC, nZVI, and GO–nZVI nanohybrids. The peak at about 10° in both GO and GO-NHS/EDC samples confirms the characteristics peak of GO, which gets suppressed by the stronger iron peaks (not observed) in the GO–nZVI nanohybrids.^37^ nZVI particles inhibit the formation of van der Waals and π–π stacking bond between the GO sheets causing the suppression of the graphitic peaks.^38,39^ The XRD pattern of GO-NHS/EDC shows three new peaks at 35.4°, 38.5° and 40.3° which were not observed for GO and can be closely attributed to the diffraction peaks of succinimide (JCPDS card no. 00-007-0745). The absence of diffraction peaks of NHS (JCPDS card no. 00-036-1688) suggests no residual NHS is present in GO-NHS/EDC. This indicates the successful formation of NHS active ester on GO surface confirming activation of GO surface by proper crosslinking of NHS and EDC.^40^ Also, the indicative peak of GO in GO/NHS-EDC is broader than in GO maybe due to some degree of aggregation of GO-NHS/EDC and NHS/EDC bonding on the GO surface.^41^ The samples of nZVI and GO–nZVI hybrids show strong characteristic peaks for nZVI at 44.75° and 82.55°.^42,43^ Moreover, the characteristic peaks of iron-oxide at 2*θ* = 30.11°, 35.43°, 43.22°, 53.62°, 57.14°, 62.78°, 65.03° and 74.24° for nZVI and GO–nZVI hybrids indicate the presence of Fe_3_O_4_*i.e.*, the oxide-shell around nZVI particles observed in HRTEM and HAADF images.^5,42,44^ XRD patterns of GO-NHS/EDC and GO–nZVI (1 : 1) showed two peaks at 35.4° and 40.3° that can be attributed to the part of NHS/EDC crosslinking. However, these peaks cannot be seen in other hybrids as they are suppressed by high iron concentration.

### Thermal stability of GO–nZVI nanohybrids

Thermal properties and stabilities of GO, GO-NHS/EDC, and GO–nZVI nanohybrids observed *via* TGA are presented in Fig. 3b. The TGA curve of GO showed that it lost about 68 wt% at three steps. At the first step which was up to 100 °C, GO lost about 11 wt% mainly due to evaporation of water molecules.^45^ The second step was from 100 to 300 °C where about 47.5 wt% of weight loss happened due to the decomposition of labile oxygen functional groups like carboxyl groups.^45^ The less steep slope of weight loss above 300 °C (15.2 wt%) can be attributed to the removal of more stable oxygen functional groups, and the release of CO and CO_2_ during char formation.^45–48^ Additional weight loss in the case of GO-NHS/EDC (at ∼400–600 °C) can be ascribed to the decomposition of amino silane coupling agent.^49,50^ The increasing residual ash content for GO–nZVI (1 : 1), GO–nZVI (1 : 5), and GO–nZVI (1 : 10) *i.e.*, 65%, 87%, and 94% of their initial mass, respectively, suggest increasing nZVI particle loading for these nanohybrids.^51^

### Molecular vibrations and functional groups of GO–nZVI nanohybrids

**Fig. 4:**
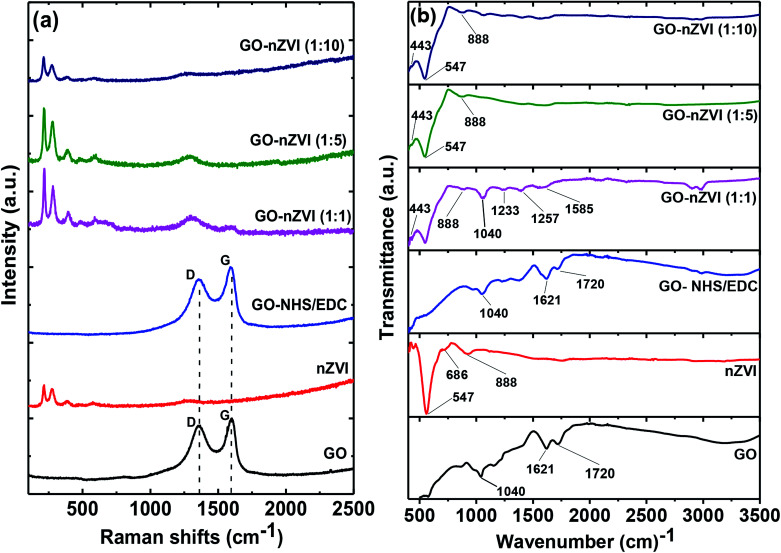
(a) Raman spectra of GO, nZVI, GO-NHS/EDC, GO–nZVI (1 : 1), GO–nZVI (1 : 5), and GO–nZVI (1 : 10), and (b) FTIR spectra of GO, nZVI, GO-NHS/EDC, GO–nZVI (1 : 1), GO–nZVI (1 : 5), and GO–nZVI (1 : 10).

Raman spectra of GO, nZVI, GO-NHS/EDC, and GO–nZVI nanohybrids are presented in Fig. 4a. The G band corresponding to ordered sp^2^ carbon atoms and the D band corresponding to defects and disorders caused by sp^3^ carbon atoms in graphitic sheets can be observed at ∼1607 cm^−1^ and ∼1368 cm^−1^, respectively in the samples containing GO.^52^ For nZVI particles, the Raman shifts are observed at 218, 283, 398, 487, 606, and 1315 cm^−1^ due to Fe–O stretching vibration.^53^ The intensity ratio of D to G (*I*_D_/*I*_G_) can be used to measure the defect and disorder degree in the graphitic matrix.^54^ The *I*_D_/*I*_G_ values of GO and GO-NHS/EDC were 0.90 and 0.84, respectively, indicating lower defects in the graphitic matrix for cross-linked GO compared to GO. The presence of characteristic Raman peaks of iron in the nanohybrids samples further confirms the impregnation of iron nanoparticles onto the graphene sheets.^30^ In nanohybrids, the graphitic carbon peaks were flushed away due to high coverage of nZVI particles on the graphene sheets. As a result, *I*_D_/*I*_G_ values of these nanohybrids were not quantifiable. The obtuse Raman peak at ∼1315 cm^−1^ observed in these nanohybrids, which is close to characteristic *I*_D_ graphitic peak, was actually from the Raman signal of hybridized nZVI particles.^55^

Fig. 4b shows the FTIR spectra of GO, nZVI, GO-NHS/EDC, and GO–nZVI nanohybrids. The bands at 1720, 1621, and 1040 cm^−1^ in GO and GO-NHS/EDC spectra indicate stretching vibrations from C=O, skeletal vibrations from unoxidized graphitic domains (C=C), and C–O stretching vibrations, respectively.^56^ Three new characteristic bands at 1585 cm^−1^ (–NH primary amide), 1257 cm^−1^ (C–N stretch of aromatic amines), and 1233 cm^−1^ (C–N stretch of aliphatic amines) appeared after crosslinking of GO with NHS/EDC and also in GO–nZVI nanohybrids.^57^ In the nZVI spectrum, the bands at 443, 547, and 686 cm^−1^ can be assigned to Fe–O bonds, and the band at 888 cm^−1^ can be assigned to the Fe–O–H bending vibration of α-FeOOH particle.^58–60^ These characteristic iron signals can also be seen in GO–nZVI nanohybrids indicating the presence of nZVI particles in the synthesized nanohybrids.^59^ The iron signals weakened in the case of nanohybrids compared to bare nZVI, which can be attributed to the presence of GO support in the nanohybrids. These results indicate the successful conjugation of GO and nZVI.

### Mechanism of GO–nZVI conjugation

Along with the high affinity of nZVI towards amine functional groups,^21,22^ amine groups are nucleophile meaning that they can donate a pair of electron to form new covalent bonds.^61^ Thus, GO-NHS/EDC, which contains amine groups (as found in FTIR spectra, Fig. 4b), is suitable for reacting with nZVI or FeOOH. A previous study showed nZVI with oxide shell (as shown in TEM images, Fig. 1e and f) is able to conjugate with amine functionalized GO.^62^ Taking all these evidences and literature reports together, we can say that NHS/EDC was able to successfully provide sufficient amine groups capable of linking or immobilizing nZVI nanoparticles onto GO surface to produce GO–nZVI nanohybrids.

### MB removal from aqueous solution by GO–nZVI nanohybrids

Although GO can remove MB from the aqueous solution, its separation from the solution is difficult and requires high-speed centrifugation.^63^ One of the aims of this study was to synthesize nano-adsorbents with the capability of magnetic separation from an aqueous media. Among the synthesized nanohybrids, GO–nZVI (1 : 5) and GO–nZVI (1 : 10) showed effective magnetic separation capacity, with separation from the aqueous solution between 4–5 minutes. However, the magnetic property of GO–nZVI (1 : 1) was not strong enough for quick and effective magnetic separation of the adsorbents. The light absorption spectra of MB solution before and after adsorption by using different amount of these adsorbents are presented in Fig. S6.†

Removal percentage of MB from aqueous solution using nZVI, GO–nZVI (1 : 5), and GO–nZVI (1 : 10) are presented in Table 2. Based on the results, although nZVI could remove MB, its efficiency as an adsorbent was limited, maybe due to larger particle size caused by aggregation as can be seen from HRTEM images. However, the nanohybrids had higher MB removal compared to nZVI, with enhanced MB removal with the increase of adsorbent dosage. About 78.3–99.1% of MB removal was achieved by using 0.1–1 mg mL^−1^ of GO–nZVI (1 : 5) as an adsorbent. Similarly, 58.2–92.6% MB removal was achieved by using 0.1–1 mg mL^−1^ GO–nZVI (1 : 10) as an adsorbent. However, the highest concentration of bare nZVI *i.e.*, 1 mg mL^−1^ could only remove ∼62% MB from the solution. GO–nZVI (1 : 5) showed the highest MB removal percentage suggesting that not only did it have sufficient nZVI content for easy and quick magnetic separation, but also it had optimum nZVI particle content on the GO surface that resulted in reduced sizes of nZVI particles (Fig. S2†) resisting aggregation. In order to compare the results from our study with the literature reported values of MB removal by magnetic nanoparticle adsorbents, we listed several study results in Table S1.† Although an extensive body of studies exist in the literature regarding similar MB removal processes, there are significant procedural differences among those studies (*e.g.*, different adsorbent dosages, different adsorption times) which make it difficult to compare the results across the literature. Compared to the study results presented in Table S1,† our GO–nZVI (1 : 5) nanohybrid was able to provide a high MB removal efficiency, especially within a short time period (5 min). For instance, 0.3 mg mL^−1^ of GO–nZVI (1 : 5) was able to remove 91.8% of MB from 12 ppm MB solution while 0.4 mg mL^−1^ of nZVI-bamboo^64^ was able to remove 92.3% MB from 10 ppm MB solution in 120 min. This provides the evidence for the rapid removal of contaminants using the synthesized GO–nZVI nanohybrids in this study.

**Table 2:** Removal efficiency of GO, nZVI and GO/nZVI on MB removal from aqueous solution

Adsorbent dosage (mg mL^−1^ of MB)	Removal efficiency (%)		
	nZVI	GO/nZVI (1 : 5)	GO/nZVI (1 : 10)
0.1	46.5 ± 1.6	78.3 ± 1.6	58.2 ± 1.4
0.3	57.0 ± 4.8	91.8 ± 0.7	61.9 ± 2.0
0.5	62.0 ± 3.7	95.4 ± 1.1	72.4 ± 2.2
1	61.0 ± 1.6	99.1 ± 0.4	92.6 ± 1.6

### Nanohybrids recyclability

**Fig. 5:**
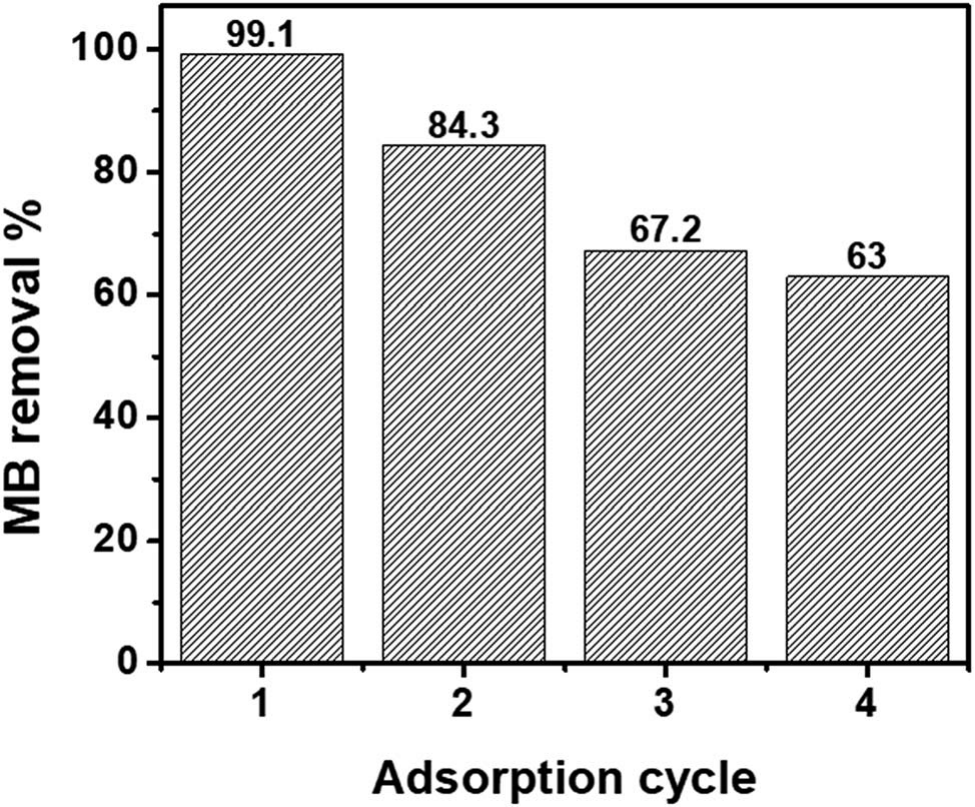
Performance of GO–nZVI (1 : 5) for MB removal after regeneration.

The ability to regenerate and reuse an adsorbent multiple times is directly related to its economic value.^65^ In previous studies, acetic acid,^66^ hydrochloric acid,^67^ methanol,^68^ and ethanol^66^ have been used for the regeneration of nZVI or magnetic GO. In this study, ethanol was selected for MB desorption from the nanohybrids as it is a non-toxic solvent.^69^Fig. 5 shows the recycling performance of the GO–nZVI (1 : 5) nanohybrid which showed the best performance for MB adsorption with 99.1% removal efficiency. After regeneration, the MB removal efficiency of GO–nZVI (1 : 5) nanohybrid reduced to 84.3%, 67.2%, and 63.0% from first to the third regeneration cycle. The sequential reduction of MB adsorption after the nanohybrids regeneration might be due to saturation of the adsorption sites by MB molecules.^70^

## Conclusions

GO–nZVI nanohybrids with different GO : nZVI ratios have been synthesized using biocompatible crosslinker NHS/EDC to provide adsorbents with superior ability for contaminant adsorption and magnetic separation, compared to nZVI only. Among the different ratios, GO–nZVI (1 : 5) exhibited the highest MB removal (up to ∼99%) with fast magnetic separation. This can be attributed to the synergistic contribution by GO and nZVI due to the non-aggregated and smaller sized nZVI. Thus, this study indicates the significance of regulating the nZVI particle loading amount in optimizing the performance of the nanohybrids. Further studies are needed to experimentally determine the biocompatibility and ecotoxicity of these newly synthesized hybrids in order to confirm its applicability as a safer-by-design nanohybrid.

## Conflicts of interest

The authors declare that they have no conflict of interests.

## Supplementary Material

RA-009-C8RA08386J-s001
