# Supplementary material for: Magnetic graphene oxide-nano zero valent iron (GO–nZVI) nanohybrids synthesized using biocompatible cross-linkers for methylene blue removal
Source: RSC Adv. 2019 Jan 8;9(2):963–73. doi: 10.1039/c8ra08386j (PMC9059533; doi:10.1039/c8ra08386j)
Supplement: RA-009-C8RA08386J-s001 [file RA-009-C8RA08386J-s001.pdf]

Electronic Supplementary Material (ESI) for RSC Advances.
This journal is © The Royal Society of Chemistry 2018

Supplementary Information for

Magnetic Graphene Oxide-Nano Zero Valent Iron (GO-nZVI) Nanohybrids Synthesized using Biocompatible Cross-linkers for Methylene Blue Removal

by

Novin Mehrabi,[a] Arvid Masud,[a] Moyosore Afolabi,[b] Hwang, J.W.,[c] Calderon Ortiz, G.A.,[c] and Nirupam Aich[a, *]

[a]*Department of Civil, Structural and Environmental Engineering, University at Buffalo, The State University of New York, Buffalo, NY 14260*

[b]*School of Civil and Environmental Engineering, Georgia Institute of Technology, Atlanta, GA 30332*

[c]*Department of Materials Science and Engineering, Center for Electron Microscopy and Analysis, The Ohio State University, Columbus, OH 43212*

Preparation for Submission

to

Journal of RSC Advances (December 17, 2018)

*Corresponding Author: Nirupam Aich, Phone: 716-645-0977, Email: nirupama@buffalo.edu

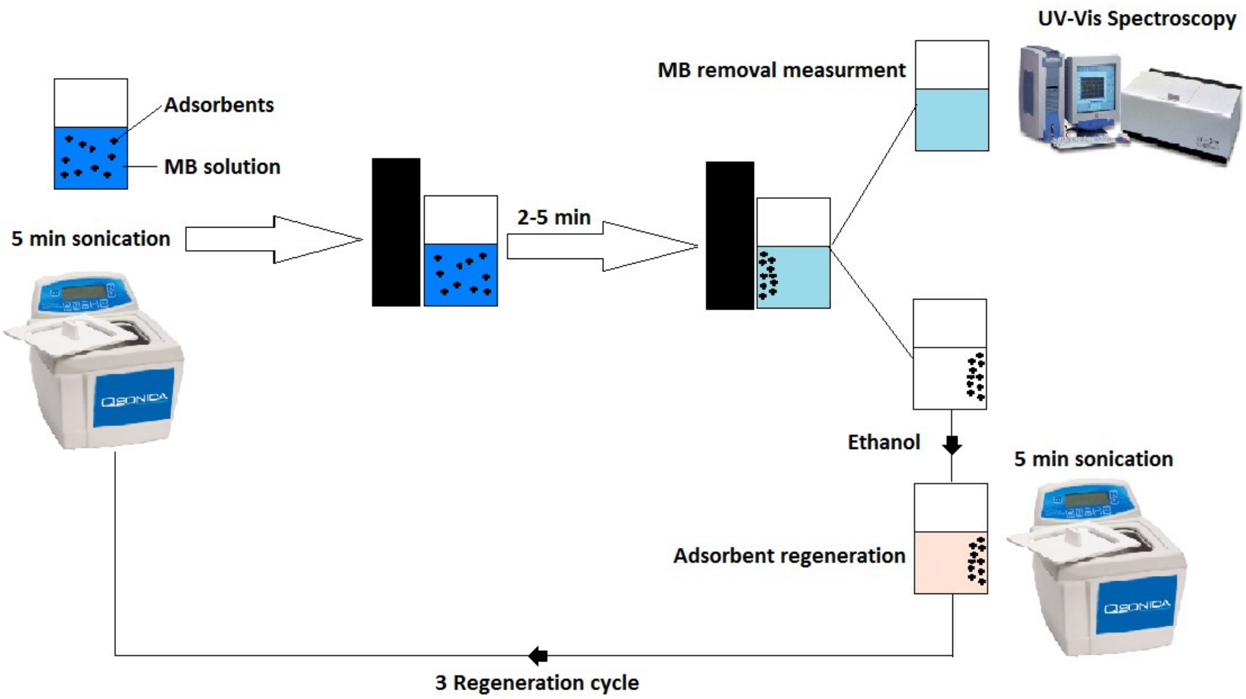

Fig. S1 Regeneration and reuse of the nanohybrids for MB adsorption.

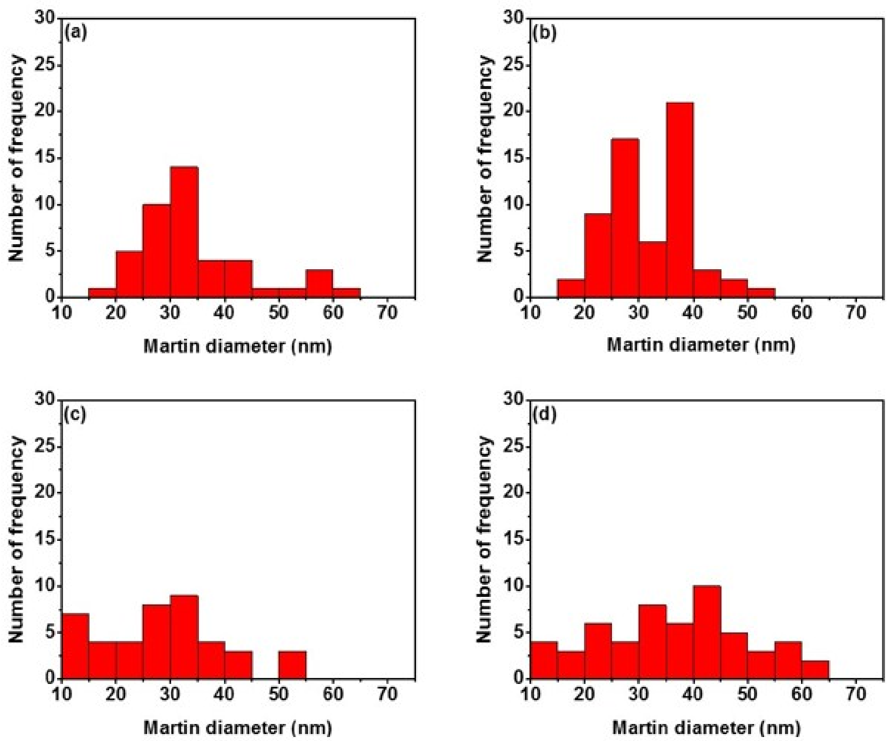

Fig. S2 Histograms of nZVI particle size distributions for (a) bare nZVI (average 38.6±17.1 nm); (b) GO-nZVI (1:1) (average 28.6±10.6 nm), (c) GO-nZVI (1:1) (average 32.6±8 nm), (d) GO-nZVI (1:1) (average 36.6±13.3 nm)

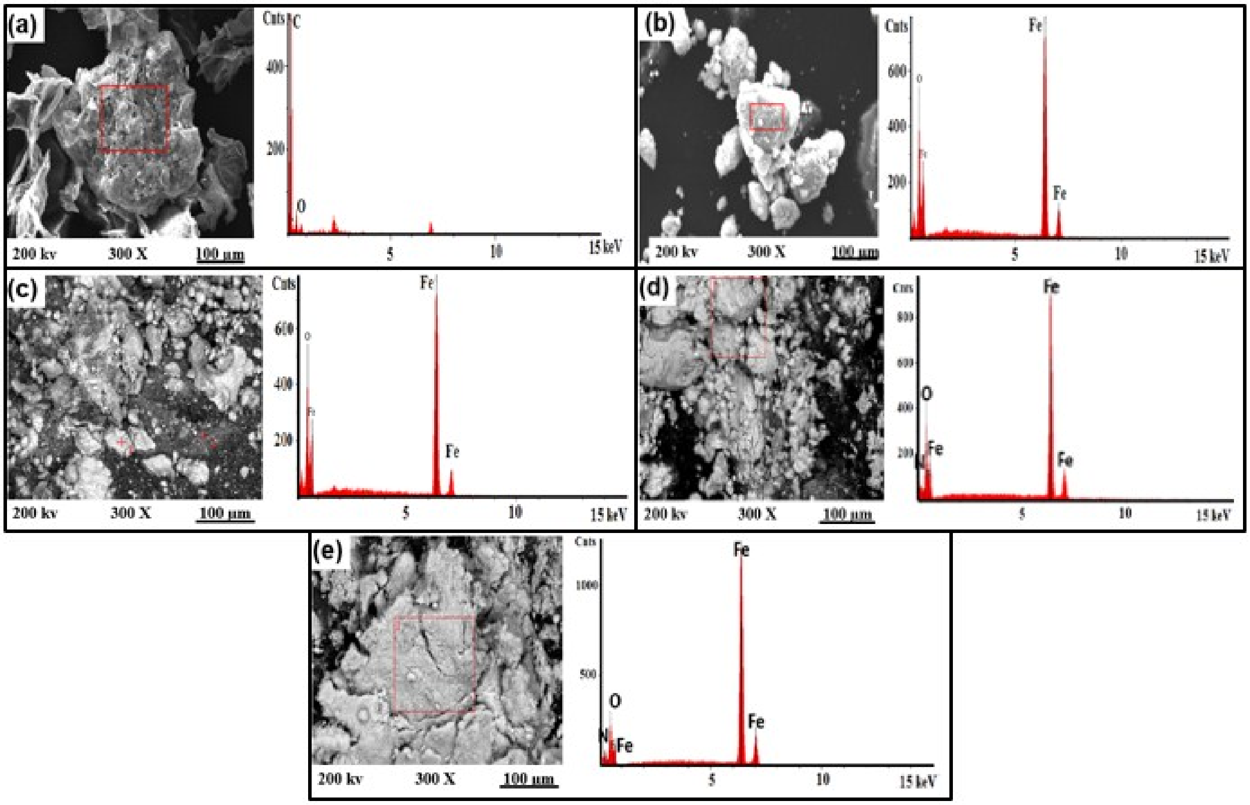

Fig. S3 SEM images and EDS analysis of (a) GO, (b) nZVI, (c) GO-nZVI (1:1), (d) GO-nZVI (1:5), and (e) GO-nZVI (1:10)

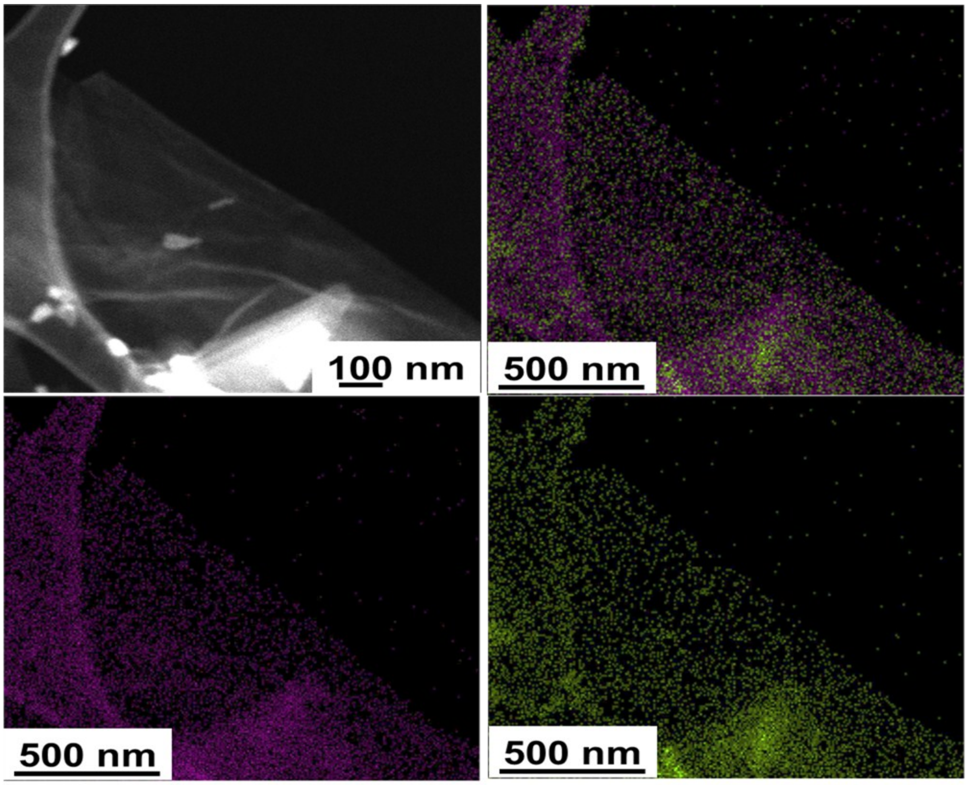

(a)

MThin Smart Quant Results (Theoretical)

Element	Weight %	Atomic %	Net Int.	Net Error%	KABFactor
C K	70.85	76.40	20.90	1.19	1
O K	29.15	23.60	11.00	2.03	0.79

C K
O K

Fig. S4(a) HAADF images with EDS mapping of single layered GO

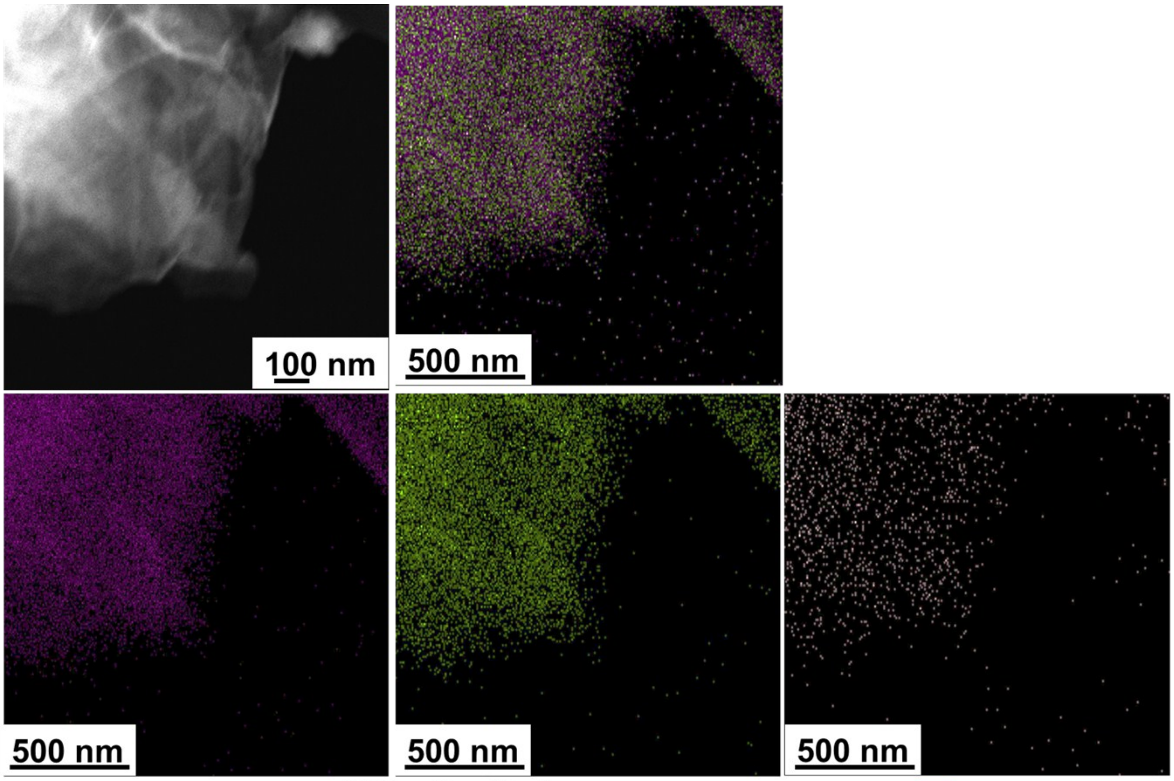

(b)

eZAF Smart Quant Results

Element	Weight %	Atomic %	Net Int.	Error %	Kratio	Z	A	F
C K	57.73	64.40	44.60	4.09	0.3925	1.0010	0.6794	1.0000
N K	1.59	1.52	0.30	99.99	0.0018	0.9988	0.1140	1.0000
O K	40.68	34.07	19.30	10.18	0.0646	0.9970	0.1592	1.0000

C K
N K
O K

Fig. S4(b) HAADF images with EDS mapping of GO-NHS/EDC

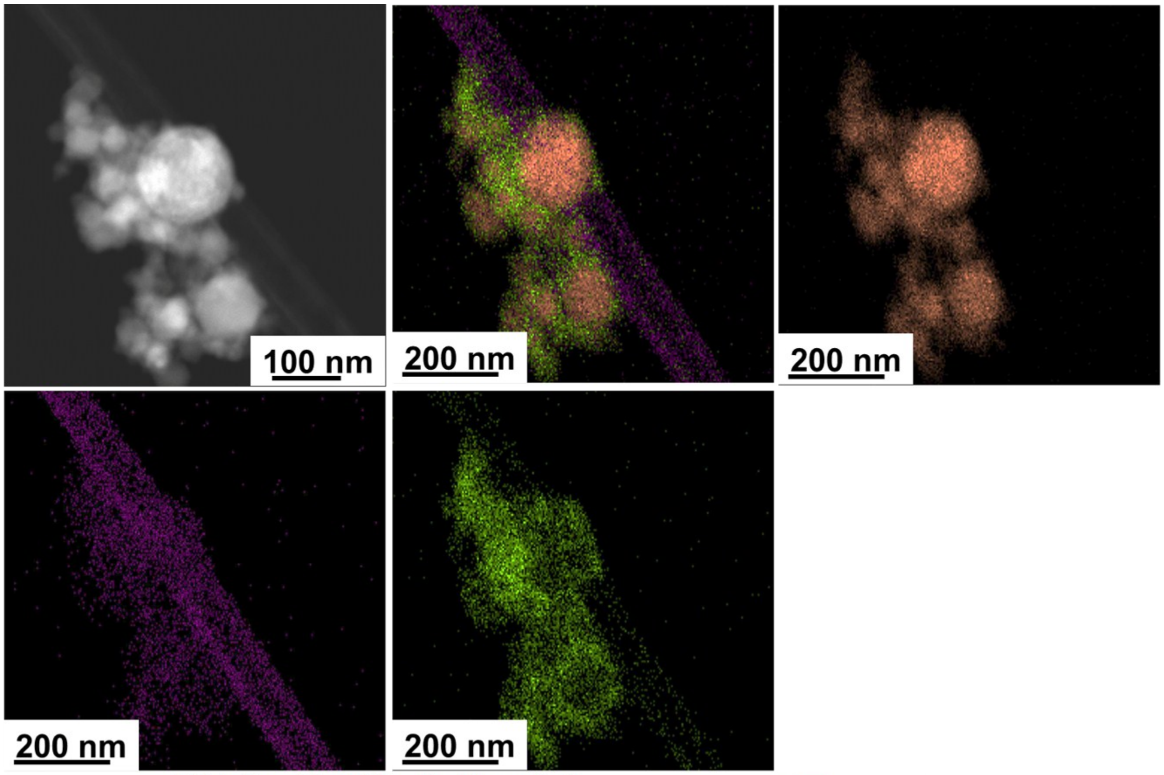

(c)

MThin Smart Quant Results (Theoretical)

Element	Weight %	Atomic %	Net Int.	Net Error%	KABFactor
C K	4.81	16.03	6.60	1.90	0.68
O K	8.79	22.00	15.40	1.10	0.53
FeK	86.40	61.97	80.80	0.44	1

Fig. S4(c) HAADF images with EDS mapping of bare nZVI

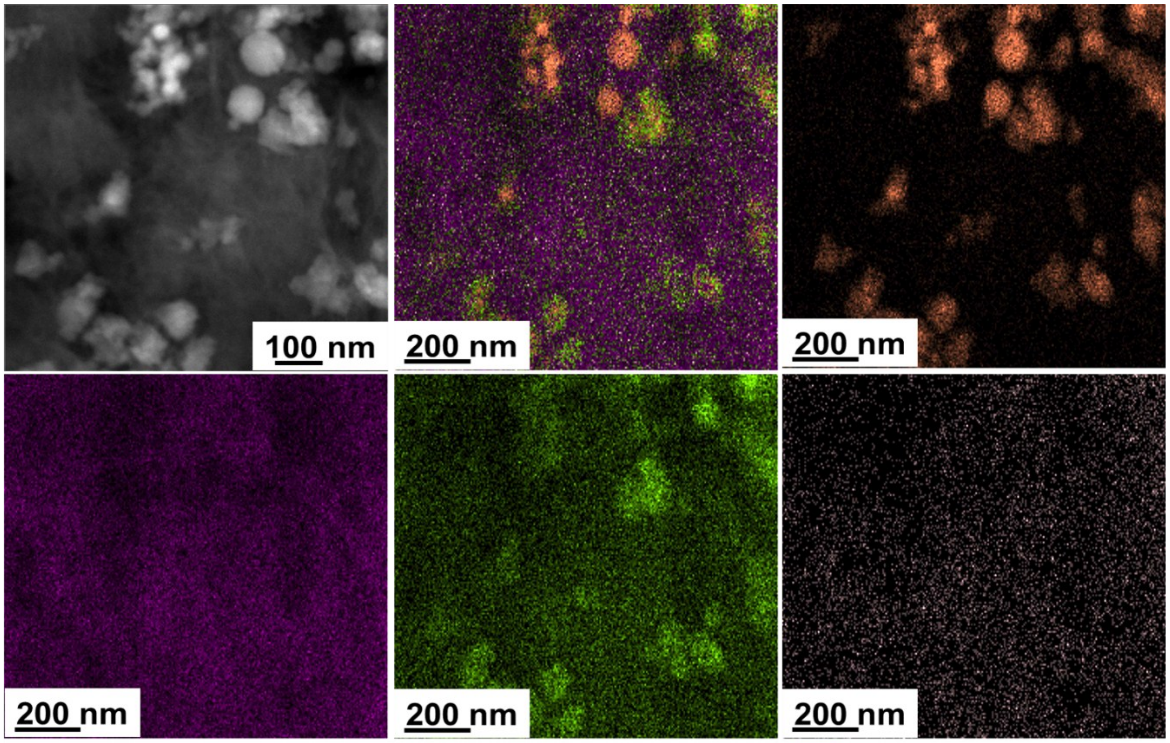

(d) **MThin Smart Quant Results (Theoretical)**

Element	Weight %	Atomic %	Net Int.	Net Error%	KABFactor
C K	54.05	72.57	243.20	0.26	1
N K	1.26	1.45	7.60	5.17	0.74
O K	18.18	18.33	104.20	0.53	0.79
FeK	26.51	7.66	80.80	0.59	1.48

C K
N K
O K
FeK

Fig. S4(d) HAADF images with EDS mapping of GO-nZVI (1:1)

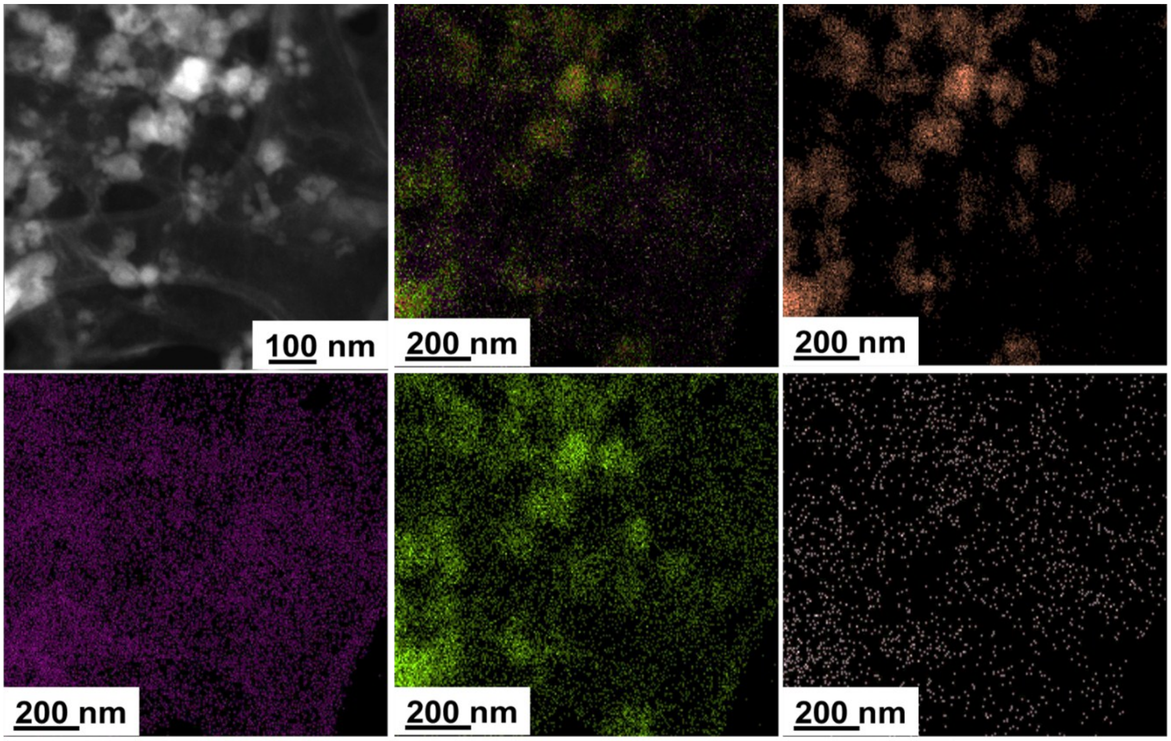

(e) MThin Smart Quant Results (Theoretical)

Element	Weight %	Atomic %	Net Int.	Net Error%	KABFactor
C K	33.87	53.81	29.00	0.90	1.27
N K	0.58	0.79	0.70	29.70	0.95
O K	27.03	32.24	29.40	1.06	1
FeK	38.52	13.16	22.30	1.35	1.88

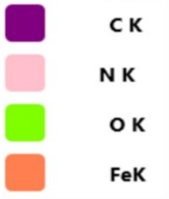

Fig. S4(e) HAADF images with EDS mapping of GO-nZVI (1:5)

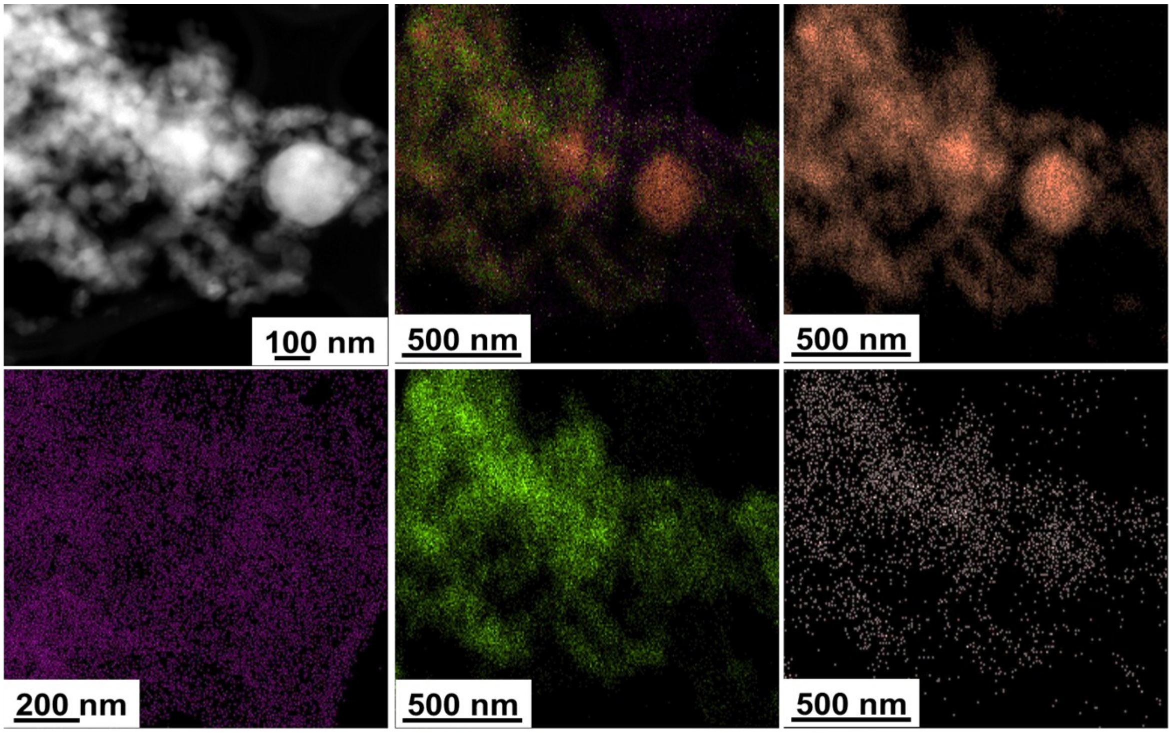

(f) MThin Smart Quant Results (Theoretical)

Element	Weight %	Atomic %	Net Int.	Net Error%	KABFactor
C K	8.92	23.23	34.10	0.83	0.68
N K	0.02	0.04	0.10	91.98	0.5
O K	18.43	36.05	89.80	0.41	0.53
FeK	72.63	40.69	188.20	0.32	1

C K
N K
O K
FeK

Fig. S4(f) HAADF images with EDS mapping of GO-nZVI (1:10)

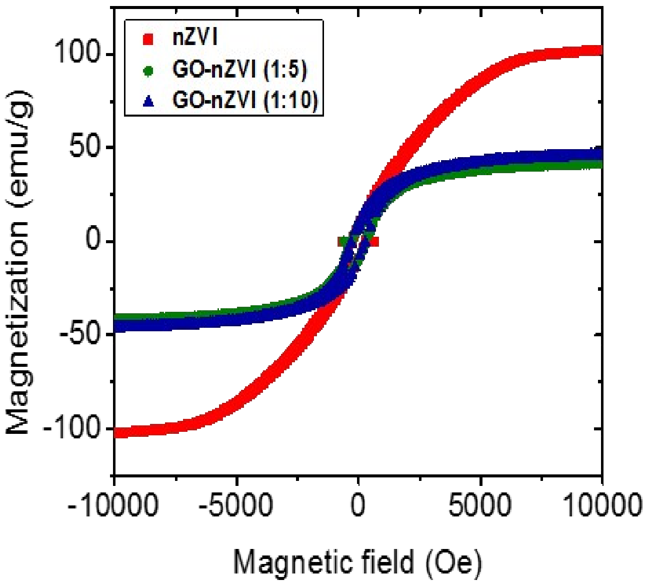

Fig. S5 Magnetic hysteresis loops of the nZVI, GO-nZVI (1:5), and GO-nZVI (1:10)

Table S1. MB removal efficiency by different magnetic adsorbents

Adsorbent	Adsorbent dosage (mg mL^{-1})	Initial MB concentration (mg L^{-1})	Adsorption time (min)	Removal Efficiency (%)	Ref.
nZVI-bamboo	0.40	10	120	92.3	[19]
Fe_3O_4-xGO (3:5)	0.28	150	180	97.5	[20]
Fe^0-Fe_3O_4-RGO	0.10	50	240	98.0	[21]
Fe_3O_4@(PAH/GO–COOH)$_2$	0.076	10	-	96.0	[22]
Fe^0 /Fe_3O_4/graphene	0.10	50	20	95.6	[23]
MMMWCNTs	0.50	20	15	~78.0	[24]
GNS/Fe_3O_4	0.4	15	2	64.0	[25]
G/Fe_3O_4	0.50	20	10	97.0	[26]
GO/nZVI (1:5)	0.10	12	5	78.3	This study
GO/nZVI (1:5)	1 .00	12	5	99.1	This study

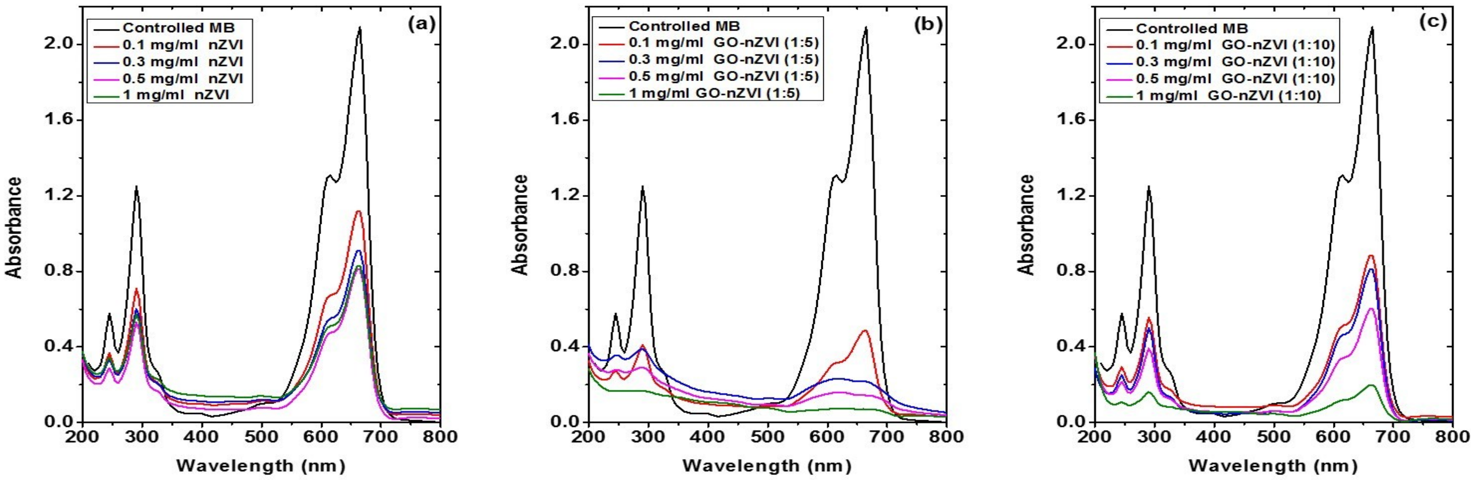

Fig. S6 Absorption spectra of controlled MB solution, and MB solutions after adsorption using (a) nZVI, (b) GO-nZVI (1:5), and (c) GO-nZVI (1:10)
